# Effect of Autophagy Regulated by Sirt1/FoxO1 Pathway on the Release of Factors Promoting Thrombosis from Vascular Endothelial Cells

**DOI:** 10.3390/ijms20174132

**Published:** 2019-08-24

**Authors:** Qian Wu, Yuting Hu, Minrui Jiang, Fanglei Wang, Guoqing Gong

**Affiliations:** Department of Pharmacology, China Pharmaceutical University, Nanjing 210009, China

**Keywords:** Sirt1/FoxO1 pathway, autophagy, vascular endothelial cell, arterial thrombosis, vWF

## Abstract

Factors promoting thrombosis such as von Willebrand factor (vWF) and P-selectin are essential for the development of atherosclerosis (AS) and arterial thrombosis. The processing, maturation and release of vWF are regulated by autophagy of vascular endothelial cells. The Sirt1/FoxO1 pathway is an important pathway to regulate autophagy of endothelial cells, therefore the Sirt1/FoxO1 pathway may be an important target for the prevention of thrombosis. We investigated the role of ox-LDL in the release of vWF and P-selectin and the expression of Sirt1 and FoxO1 by Western Blot, Flow Cytometry, ELISA, and tandem fluorescent mRFP-GFP-LC3. We found that vWF and P-selectin secretion increased and Sirt1/FoxO1 pathway was depressed in human umbilical vein endothelial cells (HUVEC) when treated with ox-LDL. Moreover, the expression of autophagy-related protein LC3-II/I and p62 increased. Then, we explored the relationship between autophagy regulated by the Sirt1/FoxO1 pathway and the secretion of vWF and P-selectin. We found that Sirt1/FoxO1, activated by the Sirt1 activators resveratrol (RSV) and SRT1720, decreased the secretion of vWF and P-selectin, which can be abolished by the autophagy inhibitor 3-MA. The expression of Rab7 increased when Sirt1/FoxO1 pathway was activated, and the accumulation of p62 was decreased. Autophagy flux was inhibited by ox-LDL and Sirt1/FoxO1 pathway might enhance autophagy flux through the promotion of the Rab7 expression. Taken together, our data suggest that by enhancing autophagy flux and decreasing the release of vWF and P-selectin, the Sirt1/FoxO1 pathway may be a promising target to prevent AS and arterial thrombosis.

## 1. Introduction

Thrombosis, one of the important causes of death in patients with cardiovascular diseases, seriously threatens the life and health of people [1]. Factors promoting thrombosis such as von Willebrand factor (vWF) and P-selectin which are stored in Weibel-Palade bodies (WPBs) of endothelial cells play important roles in the development of thrombosis as well as atherosclerosis (AS). vWF is the most abundant protein of WPBs, the processing, maturation and release of which are regulated by autophagy [2]. As an essential protein in the early stage of hemostasis, when blood vessels are damaged, vWF is released from WPBs to the blood, and combines with circulating platelets to form a loose plug [3]. In the pathologic process, vWF is deemed to be a risk factor for arterial thrombosis [4]. vWF promotes the interaction between leukocytes and vascular endothelial cells and induces infiltration of leukocytes into inflammatory tissue, which is crucial for the occurrence of AS [5]. Studies suggest that deficiency of vWF in arterial branches decreases the occurrence of AS [6]. Therefore, vWF is a promising target for the prevention of arterial thrombosis and AS. P-selectin is a member of the selectin family, which transfers to the surface of the vascular endothelial, mediates leukocyte recruitment and participates in inflammation and coagulation [7]. P-selectin, highly expressed on the surface of vascular endothelial with acute or chronic inflammation, is an ideal target for the prevention of AS [8]. Recent studies have found that autophagy of endothelial cells is associated with the occurrence and development of cardiovascular diseases. Deficiency in endothelial autophagy induces endothelial apoptosis, endothelial inflammation, and promotes atherosclerotic plaque development [9].

Autophagy is a metabolic process to degrade organelles and intracellular substances and a stress response to hunger and oxidative stress and so on, participating in the process of cell proliferation, differentiation and aging [10]. Autophagy is regulated by many signal pathways, in which the mammalian target of the rapamycin complex 1/unc-51-like autophagy activating kinase 1 (mTORC1/ULK1) pathway and the silent mating type information regulation 2 homolog 1/Forkhead box protein O1 (Sirt1/FoxO1) pathway, are the main pathways to modulate autophagy in endothelial cells. Sirt1 expression in the vasculature has effects on promoting angiogenesis and anti-inflammatory and anti-atherosclerosis through regulating transcription factors such as FoxO and NF-κB and eNOS [11]. FoxO1 is closely related to autophagy, since FoxO1 modulates the expression of many autophagy related proteins such as microtubule associated protein 1 light chain 3 (LC3), autophagy-related protein 5 (Atg5) and Beclin-1 [12].

Arterial thrombosis is usually secondary to AS [13]. With the increase of morbidity of AS, the incidence of arterial thrombosis induced by rupture of atherosclerotic plaque increases. Oxidized low-density lipoprotein (ox-LDL) is an important risk factor for AS, which damages vascular endothelium at the initial stages of AS [14]. Therefore, endothelial cells were cultured in vitro and treated with ox-LDL to simulate the process of arterial thrombosis. Considering that factors promoting thrombosis such as vWF and P-selectin secreted by endothelial cells are associated with AS, and the processing and maturation and release of vWF is regulated by autophagy, the aim of this study was to explore the function of ox-LDL on the release of vWF and P-selectin from human umbilical vein endothelial cells (HUVEC), and to assess the effect of autophagy regulated by the Sirt1/FoxO1 pathway on this process.

## 2. Results

### 2.1. Ox-LDL Decreased the Viability and Increased the Release of vWF and P-selectin as well as the Expression of LC3-II/I and p62 and Depressed the Sirt1/FoxO1 Pathway of HUVEC

**Figure 1:**
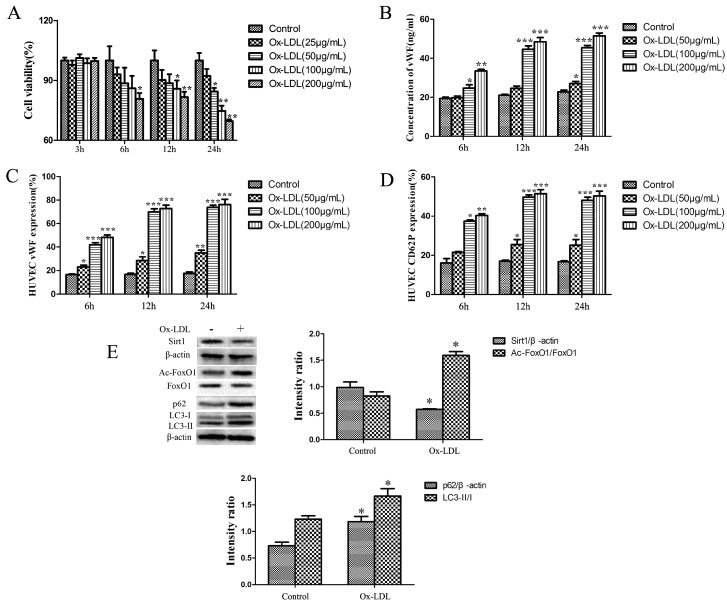
(**A**) Viability of HUVEC treated with different concentrations of ox-LDL. (**B**) Concentration of von Willebrand factor (vWF) in media. (**C**) Expression of vWF on cytomembrane. (**D**) Expression of P-selectin on cytomembrane. (**E**) Expression of Sirt1, FoxO1, LC3-II/I and p62. Values are presented as the means ± SD (MTT and ELISA assay: *n* = 6, flow cytometry and Western Blot: *n* = 3). Compared with control groups at each time point, * *p* < 0.05, ** *p* < 0.01, *** *p* < 0.001.

Cell viability of HUVEC treated with various concentrations of ox-LDL for different times is summarized in Figure 1A. Viability of HUVEC was decreased with the increase of time and the concentrations of ox-LDL compared with the control group.

The release of vWF and P-selectin from HUVEC treated with different concentrations of ox-LDL for different times is shown in Figure 1B–D. Ox-LDL dose-dependently and time-dependently increased the secretion of vWF and P-selectin. There was no significant difference between the release of vWF and P-selectin from HUVEC treated with 100 or 200 μg/mL for 12 or 24 h.

To determine the effects of ox-LDL on Sirt1/FoxO1 pathway and the expression of autophagy related proteins, the expression of Sirt1, FoxO1, Ac-FoxO1, LC3-II/I and p62 was analyzed. A total of 100 μg/mL ox-LDL treated HUVEC for 12 h decreased the expression of Sirt1 and the deacetylation of Ac-FoxO1 significantly. Additionally, the expression of LC3-II/I and p62 was increased significantly in HUVEC treated with ox-LDL, as compared with the control group (Figure 1E).

### 2.2. RSV or SRT1720 Increased the Viability of HUVEC and the Sirt1/FoxO1/Rab7 Pathway and Decreased the Accumulation of p62

**Figure 2:**
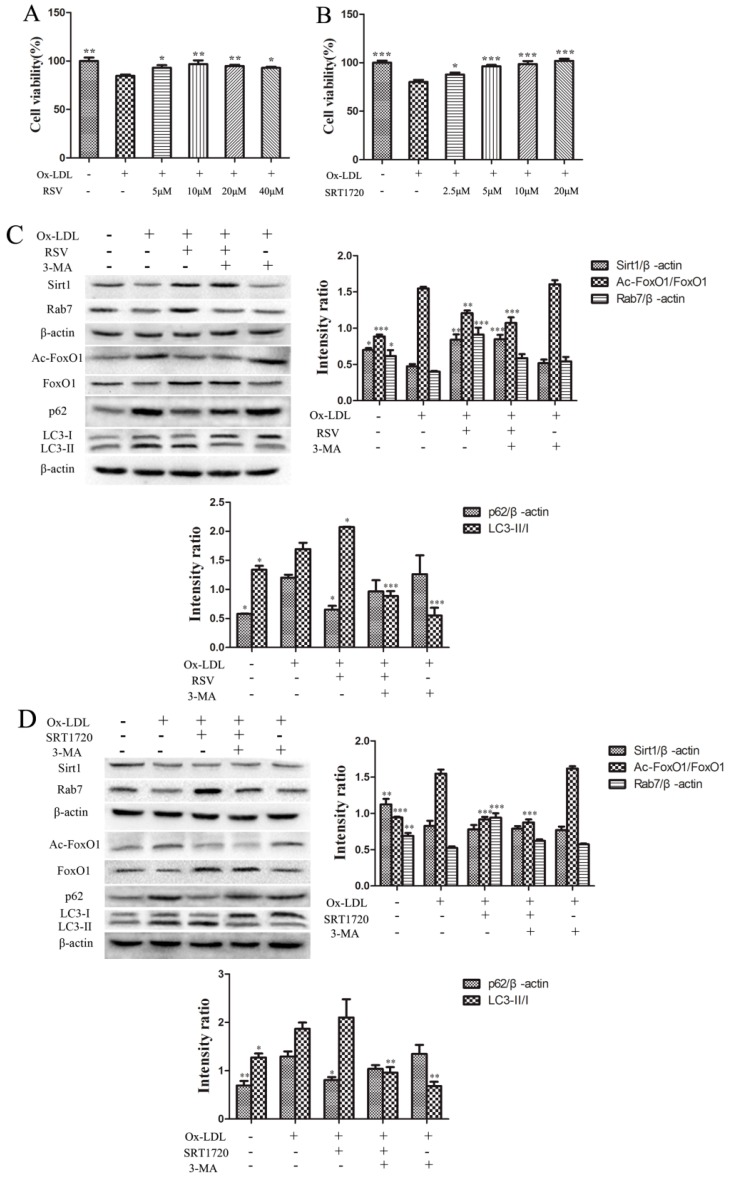
The viability of HUVEC treated with RSV (**A**) or SRT1720 (**B**). (**C**) Expression of Sirt1, FoxO1, Ac-FoxO1, LC3-II/I and p62 in HUVEC treated with RSV. (**D**) Expression of Sirt1, FoxO1, Ac-FoxO1, LC3-II/I and p62 in HUVEC treated with SRT1720. Values are presented as the means ± SD (MTT assay: *n* = 6; Western Blot: *n* = 3). Compared with the ox-LDL group, * *p* < 0.05, ** *p* < 0.01, *** *p* < 0.001.

An MTT assay was performed to determine the effects of RSV or SRT1720 on the viability of HUVEC treated with 100 μg/mL ox-LDL for 12 h. As shown in Figure 2A, the viability of RSV treated groups was increased significantly, and there was no significant difference between the 10 and 20 μM groups. As shown in Figure 2B, the viability of SRT1720 treated groups was increased dose-dependently, and there is no significant difference between 5, 10 and 20 μM groups.

To further determine the effects of the Sirt1/FoxO1 pathway on autophagy in HUVEC treated with ox-LDL, we cultured HUVEC with 100 μg/mL ox-LDL and 10 μM RSV or 5 μM SRT1720 for 12 h. The expression of Sirt1, FoxO1, Ac-FoxO1, Rab7, LC3-II/I and p62 in HUVEC treated with RSV or SRT1720 was determined by Western Blot. As shown in Figure 2C, the expression of Sirt1, Rab7 and the deacetylation of Ac-FoxO1 were increased, and the expression of p62 was decreased in HUVEC treated with RSV. The autophagy inhibitor 3-MA abolished the effects of RSV. As shown in Figure 2D, the expression of Rab7 and deacetylation of Ac-FoxO1 was increased significantly, and the expression of p62 was decreased in SRT1720 treated group, as compared with the ox-LDL group, but 3-MA inhibited the effects of SRT1720. The results suggested that RSV or SRT1720 activated the Sirt1/FoxO1 pathway to increase the expression of Rab7 and decrease the accumulation of p62, which was induced by ox-LDL.

### 2.3. RSV or SRT1720 Decreased the Release of vWF and P-selectin from HUVEC Treated with Ox-LDL

**Figure 3:**
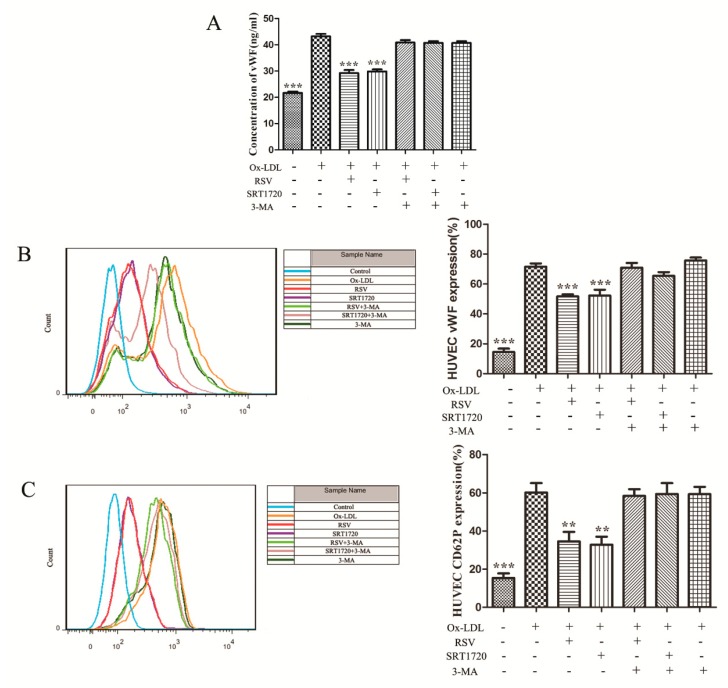
The release of vWF to extracellular (**A**) and the secretion of vWF (**B**) and P-selectin (**C**) to cytomembrane. Values are presented as the means ± SD (ELISA assay: *n* = 6, flow cytometry: *n* = 3). Compared with the ox-LDL group, ** *p* < 0.01, *** *p* < 0.001.

As autophagy was related to the secretion of vWF, we further examined whether the Sirt1/FoxO1 pathway, which regulates autophagy, had an influence on the secretion of vWF and P-selectin induced by ox-LDL. vWF secretion was detected by ELISA assay and flow cytometry, and P-selectin secretion was detected by flow cytometry. Compared to the ox-LDL group, RSV or SRT1720 significantly decreased the secretion of vWF to the extracellular and cytomembranes (Figure 3A,B). P-selectin secretion to cytomembrane was also decreased significantly by RSV or SRT1720 (Figure 3C), and the effect of RSV or SRT1720 was abolished by 3-MA. These results suggested that the effect of RSV or SRT1720 decreasing the release of vWF and P-seletin was related to autophagy.

### 2.4. RSV or SRT1720 Increased the Autophagy Flux in HUVEC Treated with ox-LDL

**Figure 4:**
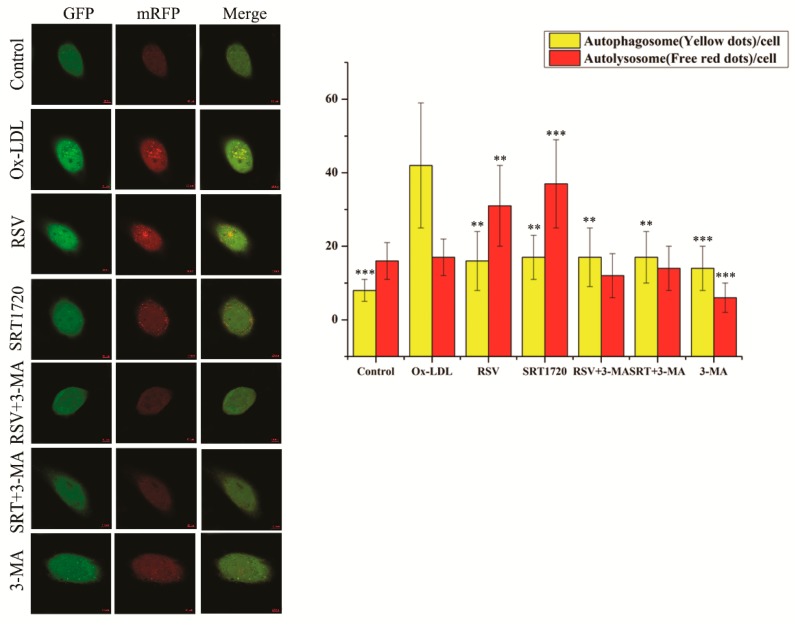
HUVEC were transfected with mRFP-GFP-LC3 adenoviral particles (MOI = 50) for 48 h, during which HUVEC were cultured with 100 μg/mL ox-LDL and 10 μM RSV or 5 μM SRT1720 for 12 h. The formation of autophagosome (yellow fluorescence) and autolysosome (red fluorescence) was detected by confocal microscopy (Scale bar, 10μm, 40 × 10 magnification). Values are presented as the means ± SD (*n* = 15). Compared with the ox-LDL group, ** *p* < 0.01, *** *p* < 0.001.

To further investigate the effect of the Sirt1/FoxO1 pathway on the autophagy flux in HUVEC treated with ox-LDL, we used mRFP-GFP-LC3 adenoviral particles (MOI = 5zdlq0) to transfect the HUVEC for 48 h, during which HUVEC were treated with 100 μg/mL ox-LDL and 10 μM RSV or 5 μM SRT1720 for 12 h. Then, we observed the formation of autophagosome (yellow fluorescence) and autolysosome (red fluorescence) by confocal microscopy. Increased autophagy flux is when there are more red fluorescent dots than yellow fluorescent dots. As shown in Figure 4, ox-LDL significantly increased the formation of autophagosome, as compared to the control group, and RSV or SRT1720 significantly decreased the accumulation of autophagosome, but increased the formation of autolysosome. The effect of RSV or SRT1720 was inhibited by 3-MA. These results suggested that ox-LDL decreased the autophagy flux, and that RSV or SRT1720 increased the autophagy flux which was inhibited by ox-LDL.

### 2.5. Effect of Gene Silencing Rab7 on Autophagy-Related Proteins

**Figure 5:**
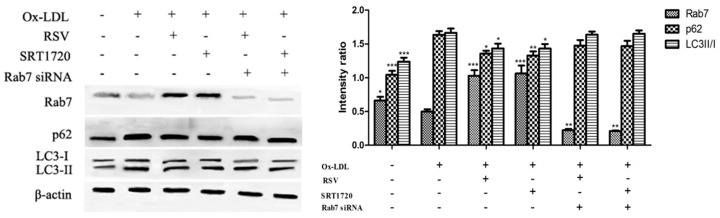
Effect of gene silencing Rab7 on the expression of Rab7, LC3-II/I and p62. HUVECs were transfected with the Rab7 siRNA for 24 h incubated with 100 μg/mL ox-LDL and 10 μM RSV or 5 μM SRT1720 for 12 h. Expression of Rab7, LC3-II/I and p62 in HUVECs treated with RSV or SRT1720 was determined by Western blot. Values are presented as the means ± SD (*n* = 3). Statistical significance between the ox-LDL treated group and other groups was determined by one-way ANOVA: Compared with the ox-LDL group, * *p* < 0.05, ** *p* < 0.01, *** *p* < 0.001.

To confirm the role of Rab7 on the effect of the Sirt1/FoxO1 pathway on autophagy in HUVEC treated with ox-LDL, we used Western blot to determine the expression of Rab7, LC3-II/I and p62 in HUVEC treated with RSV or SRT1720 after gene silencing of Rab7. As shown in Figure 5, expression of Rab7 was increased in HUVECs treated with RSV or SRT1720, and expression of p62 was decreased. The results suggested that RSV or SRT1720 activated the Sirt1/FoxO1 pathway, increased Rab7 expression and reduced ox-LDL-induced accumulation of p62, and that silencing Rab7 eliminated this effect.

### 2.6. Effect of Gene Silencing Rab7 on the Inhibition Secretion of vWF and P-selectin by Activated Sirt1/FoxO1 Pathway

**Figure 6:**
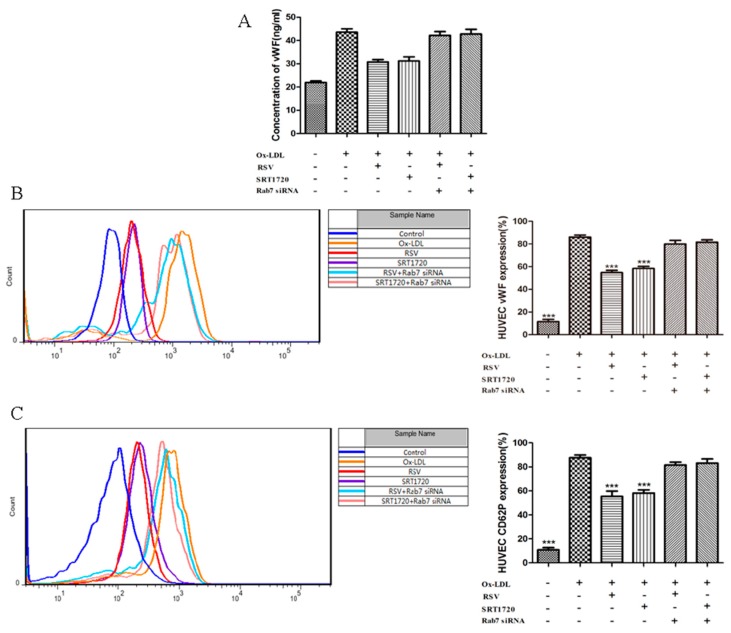
Effect of gene silencing Rab7 on the inhibition of vWF and P-selectin secretion by Sirt1/FoxO1 pathway activation. HUVECs were transfected with Rab7 siRNA for 24 h and incubated with 100 μg/mL ox-LDL and 10 μM RSV or 5 μM SRT1720 for 12 h. The secretion of vWF to extracellular (**A**) was determined by ELISA assay, and the secretion of vWF (**B**) and P-selectin (**C**) to cytomembrane was detected by flow cytometry. Values are presented as the means ± SD (ELISA assay: *n* = 6, flow cytometry: *n* = 3). Statistical significance between the ox-LDL treated group and other groups was determined by one-way ANOVA: Compared with the ox-LDL group, *** *p* < 0.001.

To further investigate the role of Rab7 in the anti-thrombotic process of the Sirt1/FoxO1 pathway, we examined whether silencing of Rab7 can affect the inhibition of ox-LDL-induced vWF and P-selectin secretion by the Sirt1/FoxO1 pathway. vWF secretion was measured by ELISA assay and flow cytometry, and P-selectin secretion was detected by flow cytometry. Compared with the ox-LDL group, RSV or SRT1720 significantly reduced the secretion of vWF to the extracellular and cytomembrane (Figure 6A,B), and RSV or SRT1720 also significantly decreased the secretion of P-selectin to the cytomembrane (Figure 6C). Silencing Rab7 can eliminate the effects of RSV or SRT1720. These results suggested that Rab7 plays an important role in the release of vWF and P-seletin influenced by Sirt1/FoxO1.

### 2.7. Effect of Gene Silencing Rab7 on Autophagic Flux in HUVECs by Activation of Sirt1/FoxO1 Pathway

**Figure 7:**
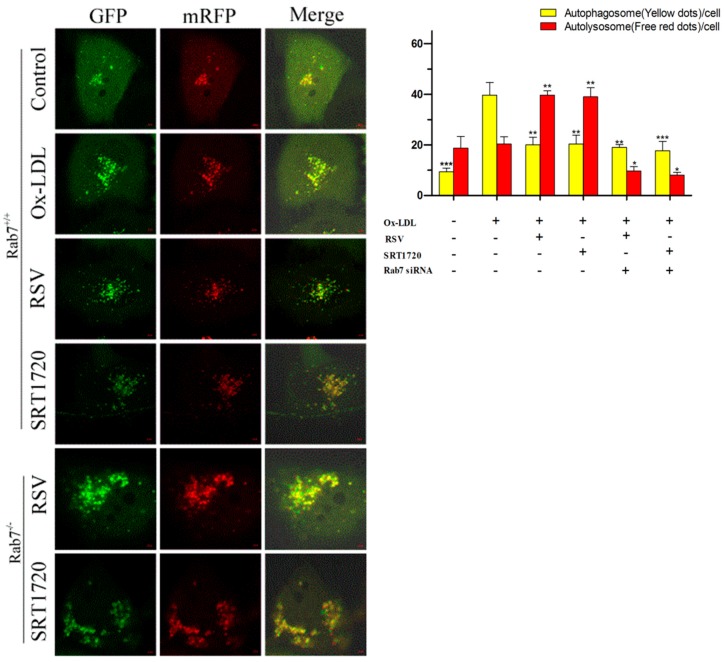
Effects of RSV or SRT1720 on autophagy flux in HUVEC treated with ox-LDL. HUVEC were transfected with mRFP-GFP-LC3 adenoviral particles (MOI = 50) for 48 h and transfected with Rab7 siRNA for 24 h, during which HUVEC were cultured with 100 μg/mL ox-LDL and 10 μM RSV or 5 μM SRT1720 for 12 h. The formation of autophagosome (yellow fluorescence) and autolysosome (red fluorescence) was detected by confocal microscopy (Scale bar, 5 μm, 63 × 10 magnification). Values are presented as the means ± SD (*n* = 15). Statistical significance between the ox-LDL treated group and other groups was determined by one-way ANOVA: Compared with the ox-LDL group, * *p* < 0.05, ** *p* < 0.01, *** *p* < 0.001.

To further investigate the role of Rab7 in the process of the effect of the Sirt1/FoxO1 pathway on autophagy flux in HUVECs treated with ox-LDL, we transfected HUVEC for 48h with mRFP-GFP-LC3 adenovirus particles (MOI = 50) and transfected with Rab7 siRNA for 24 h, during which time 100 μg/mL ox-LDL and 10 μM RSV or 5 μM SRT1720 were treated with HUVEC for 12 h. Then, we observed the formation of autophagosome (yellow fluorescence) and autolysosome (red fluorescence) by confocal microscopy. As shown in Figure 7, ox-LDL significantly increased the formation of autophagosome compared with the control group, and RSV or SRT1720 significantly decreased the formation of autophagosome but increased the formation of autolysosome. Silencing Rab7 could inhibit the effects of RSV or SRT1720 on autophagosomes. These results suggested that Rab7 plays an important role in increasing the autophagy flux decreased by ox-LDL of RSV or SRT1720.

## 3. Discussion

**Figure 8:**
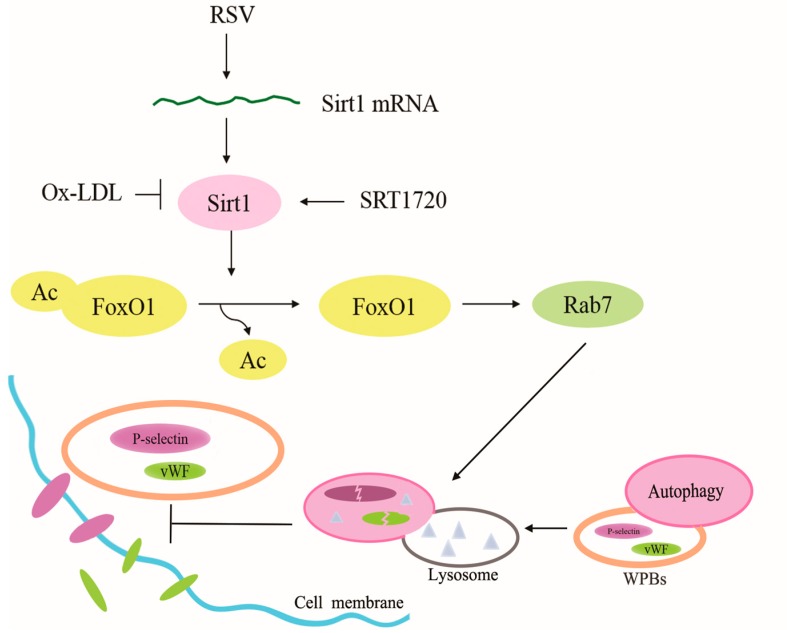
Mechanism of Sirt1/FoxO1 pathway regulating autophagy to modulate the release of vWF and P-selectin (Arrow: activation/increase; T arrow: inhibition).

The present study suggests that ox-LDL increased the secretion of vWF and P-selectin from HUVEC, which was related to the effect of ox-LDL on depressing the Sirt1/FoxO1 pathway and autophagy flux in HUVEC. Treatment with RSV or SRT1720, which is a Sirt1 activator, activated the Sirt1/FoxO1 pathway and increased the expression of Rab7 and decreased the accumulation of p62 to increase the autophagy flux in HUVEC treated with ox-LDL. Given that the release of vWF and P-selectin was inhibited by RSV or SRT1720, which was abolished by 3-MA, the function of Sirt1/FoxO1 in the increase of autophagy flux was likely to explain its depressive effect on the release of vWF and P-selectin (Figure 8).

Ox-LDL is one of the most important inducers of AS, up-regulating the expression of adhesion molecules such as P-selectin [15]. P-selectin is a pro-inflammatory factor stored in WPBs of vascular endothelial cells, playing an important role in the development of arterial thrombosis as well as venous thrombosis [16]. As an important protein involved in coagulation, vWF also participates in the development of thrombosis and AS [17]. Given that vWF and P-selectin are both stored in and secreted from WPBs, the relationship between ox-LDL and the release of vWF and P-selectin was investigated. Our experiments evidenced that ox-LDL induced the secretion of P-selectin as well as vWF.

Sirt1/FoxO1 pathway is an important pathway to regulate autophagy in vascular endothelial cells [18]. As the main downstream target of Sirt1, FoxO1 plays critical roles in cell stress adaption [19] and directly regulates the transcription of autophagy-related genes by binding to the promoter regions [20]. It has been reported that autophagy induced by the Sirt1/FoxO1 pathway is beneficial to the survival of cardiac myocytes lacking energy [21] and Sirt1 expression is depressed in mononuclear macrophage treated with ox-LDL, and ox-LDL can reduce the expression of Sirt1 by inhibiting LOX-1 that activates Sirt1 [22]. In consideration of vWF secretion being related to autophagy and that the Sirt1/FoxO1 pathway is important in regulating autophagy in vascular endothelial cells, the link between the Sirt1/FoxO1 pathway and the secretion of vWF and P-selectin was explored.

RSV, a natural polyphenol extracted from red wine, improves the transcription of Sirt1 mRNA to increase the expression of Sirt1 protein [23]. SRT1720, as a specific activator of Sirt1, exerts auxiliary allosteric activation by binding to the N-terminal of Sirt1 and using hydrophobic residues of substrates (or intracellular factors) to reduce the Michaelis constant of substrates, thereby increasing the activity of Sirt1 [24]. The present study suggests that the increase of vWF and P-selectin secretion induced by ox-LDL is due to the depression of Sirt1/FoxO1 pathway, and activating this pathway by RSV or SRT1720 is sufficient to decrease the release of vWF and P-selectin. The autophagy inhibitor 3-MA inhibits the effects of RSV and SRT1720, suggesting that RSV and SRT1720 inhibition of thrombotic factors release may be associated with autophagy.

Autophagy includes two processes, the formation of autophagosome and autolysosome, that autophagosome engulfs proteins or organelles and degrades these contents through fusing with lysosomes. During the fusion of autophagosome and lysosome, Rab7, the small GTP-binding protein, is essential. Rab7 recruits on the membrane of theautophagosome and mediates the fusion with the lysosome [25]. The inhibition of Rab7 has no influence on the formation of autophagosome but lysosome and depresses the fusion of them, which results in the accumulation of autophagosome [26].

LC3-II/I is a common index to the assessment of autophagy, because LC3-I is modified and processed to generate LC3-II located on the membrane of autophagosome, and then LC3-II/I increases. However, when the fusion of autophagosome and lysosome is rapid, the expression of LC3-II/I may decrease which does not account for the inhibition of autophagy. P62 is also an important indicator of autophagy, connecting LC3 with ubiquitination substrates. P62 is transferred to autophagosome with polyubiquitinated proteins and degraded in autolysosome. When autophagy is inhibited in mammalian cells, the expression of p62 increases. However, the variety of the accumulation of p62 with autophagy depends on the cell type. Accordingly, mRFP-GFP-LC3 adenovirus, an efficient tool for research of autophagy flux, is used in this study. The present study shows that ox-LDL depresses the autophagy flux of HUVEC, and that the Sirt1/FoxO1 pathway activated by RSV or SRT1720 improves the autophagy flux inhibited by ox-LDL, and also that inhibiting or knocking out Rab7 can abolish the effects of Sirt1/FoxO1. These findings suggest that autophagy flux depression is related to the increase of vWF and P-selectin secretion, and that activating the Sirt1/FoxO1 pathway is an available way to enhance autophagy flux and depress the release of vWF and P-selectin. It is worth noting that studies have shown that RSV and SRT1720 are not direct activators of Sirt1 [27,28]. As for the limitations of the research, we hope that more relevant studies will make up for them in the future.

In conclusion, our study is the first to suggest that the Sirt1/FoxO1 pathway inhibition is related to the increase of vWF and P-selectin release. Sirt1/FoxO1 pathway activated by RSV or SRT1720 improves the autophagy flux, which is depressed by ox-LDL through increasing the expression of Rab7, and decreases the release of vWF and P-selectin induced by ox-LDL. Sirt1/FoxO1 may be a potential target to prevent the development of AS and arterial thrombosis.

## 4. Materials and Methods

### 4.1. Reagents

Ox-LDL solution was purchased from Yiyuan biotechnology (Guangzhou, China); DMEM medium was obtained from HyClone (Logan, UT, USA). Fetal bovine serum was obtained from GEMINI (Woodland, CA, USA). Penicillin, streptomycin and goat anti-rabbit antibodies were obtained from YEASEN (Shanghai, China). The fluorescein isothiocyanate (FITC) anti-von Willebrand Factor antibody was purchased from Abcam (Cambridge, UK). FITC anti-human CD62P antibody was purchased from eBioscience (San Diego, CA, USA). vWF ELISA kit was purchased from Elabscience (Wuhan, China). Rabbit anti-mouse antibodies and rabbit anti-human LC3, p62 and β-actin antibodies were obtained from Wanleibio (Shenyang, China). Mouse anti-human Sirt1, FoxO1 and Rab7 antibodies and rabbit anti-human Ac-FoxO1 antibody were purchased from Santa Cruz Biotechnology (Dallas, TX, USA). The mRFP-GFP-LC3 adenoviral particles were purchased from HanBio (Shanghai, China). Rab7 siRNA was purchased from Cobioer (Nanjing, China). Lipofectamine™ RNAiMAX Transfection Reagent was purchased from Life Technologies (Grand Island, NY, USA). Rabbit anti-human Rab7 antibody was obtained from Abcam (London, UK).

### 4.2. Cell Culture

Human Umbilical Vein Endothelial Cells (HUVEC) were obtained from ATCC (Rockville, MD, USA). The cells were cultured in DMEM media supplemented with 10% FBS and penicillin-streptomycin (50 units/mL and 50 mg/mL, respectively) at 37 °C in a CO_2_ incubator with 5% CO_2_.

### 4.3. Analysis of Ox-LDL Treated HUVEC

HUVEC were cultured with a series of concentrations of ox-LDL (25, 50, 100 or 200 μg/mL) or media control for 3, 6, 12 or 24 h, viability of which was determined by MTT assay (Biosharp, Wuhan, China).

HUVEC were treated with various concentrations of ox-LDL (50, 100 or 200 μg/mL) or media control for 6, 12 or 24 h, and the media was collected to analyze the concentration of vWF released into the media by ELISA assay. HUVECs harvested by centrifugation were stained using the FITC anti-von Willebrand Factor antibody and anti-human CD62P antibody. Briefly, HUVEC (106/mL) were stained with 1 μL of FITC-Labeled vWF or P-selectin for 30 min at 22 °C in the dark. Then the cells were measured using a MACSQuant flow cytometer (MACSQuant, Bergisch Gladbach, Germany) within 1 h.

### 4.4. Analysis of the Effect of RSV or SRT1720 on the Viability of HUVEC Treated with Ox-LDL

HUVEC were cultured with 100 μg/mL ox-LDL and various concentrations of RSV (5, 10, 20 or 40 μM, Sigma-Aldrich, St. Louis, MO, USA) or SRT1720 (2.5, 5, 10 or 20 μM, Topscience, Shanghai, China). Then, the viability of cells was determined using the MTT assay.

### 4.5. siRNA Transfection

Rab7+/+HUVEC were grown to 60% confluency in a 6-well plate before siRNA transfection. siRNA against Rab7 was obtained from Cobioer (Nanjing, China) and dissolved to a stock solution with water. HUVEC were transfected with up to 10 nM siRNA against Rab7 or negative control siRNA provided by the manufacturer using Lipofectamine™ RNAiMAX (Life Technologies, Grand Island, NY, USA) as the transfection reagent following the manufacturer’s protocol for 24h before collection for analysis.

### 4.6. Determination of the Release of vWF and P-selectin from HUVEC Treated with Ox-LDL and RSV or SRT1720

HUVEC cultured with 100 μg/mL ox-LDL and 10 μM RSV or 5 μM SRT1720 or 10 mM autophagy inhibitor 3-MA (3-Methyladenine) for 12 h were harvested and stained with the FITC anti-von Willebrand Factor antibody and anti-human CD62P antibody for flow cytometric analysis. Media was collected for ELISA assay.

### 4.7. Western Blot Analysis

HUVEC treated with 100 μg/mL ox-LDL and 10 μM RSV or 5 μM SRT1720 or 10 mM 3-MA for 12 h were harvested and lysed with lysis buffer supplemented with protease inhibitors (Beyotime Biotechnology, Shanghai, China) for 30 min at 4 °C. Lysates were centrifuged at 12,000× *g* for 5 min to gather the supernatant. After determining the protein concentration, 40 μg protein was boiled for 5 min and separated on 8% or 12% SDS-polyacrylamide gel electrophoresis gels. Western blots were performed by previously described methods [29].

### 4.8. Confocal Microscopy Analysis of Autophagic Flow

HUVEC were transfected with adenovirus containing plasmids expressing tandem fluorescent mRFP-GFP-LC3 (MOI = 50) for 36 h, and then cells were treated with ox-LDL or RSV or SRT1720 or 3-MA for another 12 h. Fluorescent images were observed with confocal laser scanning microscope (LSM700, Zeiss, Oberkochen, Germany). Photographs of red and green fluorescence were taken separately, and the two were overlapped by Zen imaging software. Due to the fluorescence quenching of GFP at low pH levels found in the autolysosome, there is only red fluorescence expressing in the autolysosome. Therefore, red fluorescent dots represent autophagic lysosomes. Both green and red fluorescence express in the autophagosome, thus yellow fluorescence represents the autophagosome in the fluorescence overlapping drawings [30]. When red fluorescent dots are more than yellow, the autophagic flow is smooth and the autophagic reaction increases. When red fluorescent dots are less than yellow, the autophagic flow is inhibited.

### 4.9. Statistical Analysis

Statistical analysis was performed using SPSS 19.0 software and GraphPad Prism 5 software. Data were expressed as means ± standard deviation (SD). Shapiro–Wilk test was used to check the normality of distribution. The differences between control group and model group were compared with an unpaired Student’s *t*-test or Mann–Whitney test. Statistical significance was determined using one-way analysis of variance (ANOVA) or Mann–Whitney test to compare the model group and the other groups. Statistical significance was defined as * *p*< 0.05, ** *p*< 0.01, *** *p*< 0.001.
